# Exploring the regulatory roles of AtGLR3.4 receptors in mitochondrial stress and ROS management in Arabidopsis

**DOI:** 10.1007/s00299-025-03558-y

**Published:** 2025-07-01

**Authors:** Azime Gokce, Askim Hediye Sekmen

**Affiliations:** Department of Biology, Faculty of Science, Ege University, 35100 Bornova, Izmir Türkiye

**Keywords:** Antioxidant, Glutamate receptors, Nitric oxide, Mitochondrial stress, Rotenone

## Abstract

**Key message:**

atglr3.4.1 knockout disrupts H₂O₂-scavenging enzymes, increasing ROS and redox imbalance. This upregulates COX5B, UPOX, and UCP. AtGLR3.4.2 maintains redox homeostasis, highlighting AtGLR3.4 receptors' role in mitochondrial stress.

**Abstract:**

Glutamate receptors (iGluRs/mGluRs) play a crucial role in cognitive processes in mammals. Studies in humans have shown that the overexpression of glutamate receptors increases Ca^2^⁺ influx into the cell, leading to nitric oxide (NO) accumulation, which in turn induces mitochondrial stress. Dysregulated activity of (iGluRs/mGluRs) is linked to depression, psychosis, and neurodegenerative diseases in humans. In plants, GLRs are involved in carbon and nitrogen metabolism and seed germination. Research in Arabidopsis has shown that GLRs play a key role in generating and responding to stress signals. However, it remains unknown how GLR-mediated changes in NO levels affect mitochondria in plants. To address this question, our study investigated the effects of AtGLR3.4.1 and AtGLR3.4.2 receptors on mitochondrial stress under nitrosative stress conditions. For this purpose, we used *A. thaliana* wild type and *atglr3.4* mutants (*atglr3.4.1* and *atglr3.4.2*). To induce mitochondrial stress, we applied 80 µM Complex I inhibitor Rotenone. We examined the accumulation of reactive oxygen/nitrogen species (ROS/RNS), the effectiveness of the antioxidants responsible for their scavenging, cellular redox balance, and the expression of mitochondrial stress-related genes. The absence of AtGLR3.4.1 increased ROS accumulation by inhibiting catalase (CAT) and ascorbate peroxidase (APX) and disrupting the GSH/GSSG and NAD/NADH ratios. In *atglr3.4.2* mutants, ROS-related oxidative damage was regulated by the ascorbate–glutathione cycle. *atglr3.4.1* knockout increases the transcription of stress-related genes (COX5B, UPOX, and UCP), highlighting its role in oxidative stress management. These findings highlight AtGLR3.4 is crucial for preventing excessive ROS and redox homeostasis under mitochondrial stress responses.

## Introduction

Glutamate is the primary excitatory neurotransmitter required for mammalian nerve cells to maintain their functionality. It acts as an excitatory neurotransmitter, allowing cells, tissues or organs to be stimulated and their functions to accelerate. In mammals, glutamate receptors (iGluRs/ mGluRs) function as multimeric ion channels specialized to detect glutamate in the neurotransmission process (Armstrong et al. 1998). The binding of glutamate to these receptors causes the opening of transmembrane channels, allowing cation entry into the cell. These channels function as ligand-gated ion channels that transport cations such as Ca^2^⁺ and Na⁺ (Scatton 1993). The mammalian central nervous system contains ionotropic glutamate receptors (iGluR) and metabotropic glutamate receptors (also known as G-protein coupled receptors) that are activated by glutamate (Monaghan et al. 1989). iGluRs are involved in fast excitatory neurotransmission as well as higher-order brain functions such as learning, synaptic plasticity, and memory. They also play a critical role in fast excitatory signaling between presynaptic and postsynaptic membranes. Interestingly, iGluRs have been identified in all three domains of life and are widely distributed in animals from ctenophores to vertebrates. However, they are thought to play important roles in neuronal signaling, development, and plasticity in primitive phyla such as hydra (Pierobon 2012) and to retain these functions in more advanced nematode and vertebrate phyla. While mammalian glutamate receptors are classified as ionotropic (iGluRs) or metabotropic (mGluRs), in plants, structurally similar but functionally distinct glutamate-like receptors are consistently referred to as GLRs in the literature (Forde and Roberts 2014; Meyerhoff et al. 2005; De Bortoli et al. 2016).

Notably, iGluR-like channels have also been reported to be present in the genomes of various plant species, including Chlamydomonas, chlorophytes, mosses, ferns, gymnosperms, and angiosperms (De Bortoli et al. 2016). In the genome of the model plant *Arabidopsis thaliana*, 20 genes encoding subunits of glutamate-like receptors (AtGLRs) have been identified, and these genes have been classified into three major protein families (Clade I, Clade II, and Clade III) (Chiu et al. 2002; Davenport 2002). Plant GLRs have been shown to play critical roles in various physiological functions, such as regulation of growth and developmental processes, hormonal signaling, ion and water balance, electrical signal transmission, and activation of defense mechanisms against environmental stresses (Kang et al. 2004; Cho et al. 2009; Michard et al. 2011; Kwaaitaal et al. 2011; Lu et al. 2014; Forde and Roberts 2014; Kong et al. 2015; Iwano et al. 2015; Singh et al. 2016; Wudick et al. 2018; Vincent et al. 2017). This functional diversity reveals that GLRs make important contributions to both the developmental needs of plants and their adaptation to environmental stimuli. Furthermore, it has been shown that plants, although lacking specialized cells such as neurons, have excitable cells that can generate and transmit electrical signals (Hedrich et al. 2016). Indeed, Mousavi et al. (2013) demonstrated that AtGLRs play a critical role in transmitting electrical signals across the phloem in Arabidopsis leaves in response to caterpillar-induced wounding. These results suggest that sequence similarities between Arabidopsis GLRs and animal iGluRs suggest evolutionary conservation (Lam et al. 1998). Although plant GLRs are evolutionarily related to animal iGluRs, they do not fit neatly into AMPA or NMDA categories. However, structural and regulatory similarities have been reported. For example, GLR3.3 shows Ca^2^⁺/calmodulin-dependent regulation similar to NMDA receptors (Yan et al. 2023), and a GLR2.5 splice variant (GLR2.5c) retains key domains essential for Ca^2^⁺-permeable channel activity, indicating functional conservation (Gong et al. 2025). These findings suggest that certain structural and regulatory elements of GLRs may be evolutionarily conserved, pointing to their significance in plant-specific stress signaling pathways.

In addition to many studies related to iGluRs, Forrester et al. (2006) suggested that increased iGluR channel activation in the brain cell plasma membrane in humans may be an important mechanism in the pathogenesis of neurodegenerative diseases, leading to excessive NO production. Studies have shown that mutations in the PreP (Pre-sequence Protease) and DJ-1/PARK (Parkinson-associated gene DJ-1) genes, as well as mitochondrial stress associated with inhibition of the PDI (protein disulfide isomerase) enzyme, which promotes NO accumulation via the Glutamate receptor (NMDAR) in the brain cell membrane, cause Alzheimer's and Parkinson's in humans (Uehara et al. 2006). As Walter Manucha mentioned in his review (2017), although the etiology of neurodegenerative diseases is still unknown, increasing evidence suggests that glutamate and mitochondria are two key players in the oxidative stress process underlying these diseases. In addition, the emerging role of NO pathways associated with mitochondrial dysfunction is effective in neurotoxicity resulting from glutamate-induced apoptosis. Lam et al. (1998) discovered that, similar to human brain cell membranes, Arabidopsis cell membranes also contain NMDAR-like GLR receptors. Teardo et al. (2015) observed that in *atglr3.5* mutants, the cristae swelled, Ca^2^⁺ accumulation decreased, and plant senescence accelerated. In other words, AtGLR3.5 was identified as functioning as a Ca^2^⁺ channel. Cheng et al. (2018) further determined that the lack of activation of AtGLR3.4 led to a decrease in the amount of Ca^2^⁺ entering the cytosol. However, it remains unknown whether changes in NO content due to GLR activation induce mitochondrial stress, as observed in humans, and how this affects intracellular ROS-RNS production and the antioxidant defense system.

Therefore, in our study, we investigated the effects of the AtGLR3.4 receptor on intracellular ROS/RNS balance, the antioxidant defense system under mitochondrial stress in *Arabidopsis thaliana*. While this study does not aim to draw direct parallels with mammalian systems, we sought to investigate whether similar NO-related signaling mechanisms involving AtGLR3.4 operate within the unique physiological and stress conditions of Arabidopsis For this purpose, we compared the activity of antioxidant defense system enzymes (NADPH oxidase (NOX), superoxide dismutase (SOD), catalase (CAT), peroxidase (POX), ascorbate peroxidase (APX) and glutathione reductase (GR)), determination of ROS (H₂O₂ and O^2⋅−^) redox-related antioxidants (total glutathione ratio (GSH/GSSG), ascorbate content, glutathione-S transferase (GST), glutathione peroxidase (GPX), NAD/NADH ratio, monodehydroxyascorbate reductase (MDHAR) and dehydroxyascorbate reductase (DHAR)) and expression of mitochondrial stress-related genes in mature rosette leaves of *atglr3.4* (*atglr3.4.1* and *atglr3.4.2*) mutants and the wild-type *Arabidopsis thaliana* Columbia ecotype under mitochondrial stress induced by rotenone, a Complex I inhibitor.

## Materials and methods

### Plant material and acquisition

**Fig. 1:**
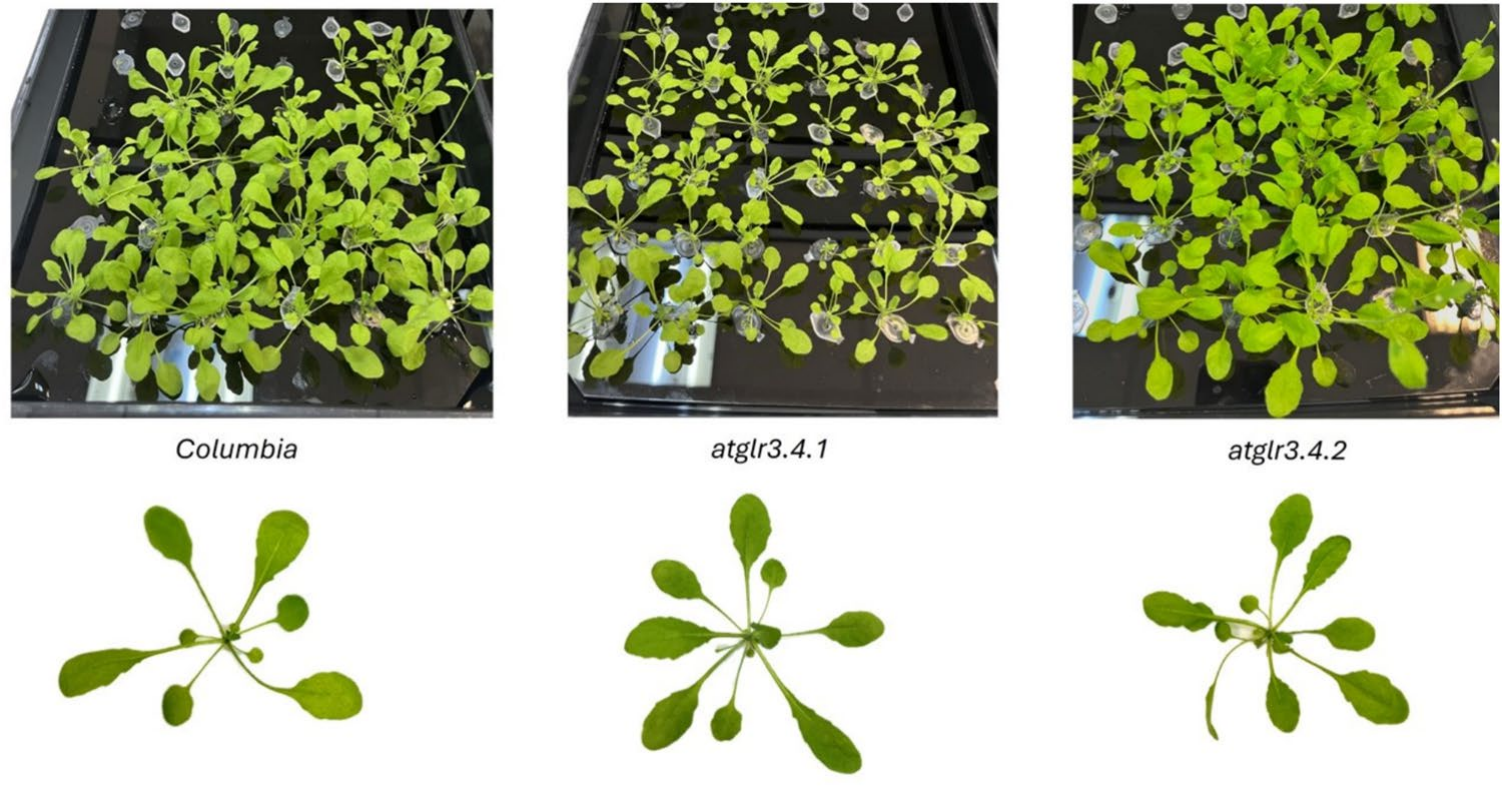
*A. thaliana* Col-0 and *atglr3.4* mutants grown in a hydroponic system

In this study, *Arabidopsis thaliana* Col. (wild type) and *Arabidopsis thaliana* GLR3.4 (glutamate receptor knockout mutants) were used as plant materials. Seeds were obtained from the Arabidopsis Biological Resource Center (ABRC). The mutant lines used were *atglr3.4.1* (SALK_079842) and *atglr3.4.2* (SALK_016904). AtGLR3.4 is expressed in all seedling tissues, showing high expression in cotyledons and young roots, while in adult plants, it is localized to mesophyll cells, vascular bundles, and hydathodes of developing leaves. Compared to its strong expression in leaves and stems, AtGLR3.4 expression is weaker in root tissues such as cortex, epidermis, and root hairs (Meyerhoff et al. 2005). Additionally, the most intense GUS staining was observed in rosette leaves (Manzoor et al. 2013). Therefore, mature rosette leaves of *A. thaliana glr3.4* mutants (*atglr3.4.1* and *atglr3.4.2*) were used in this study. These mutants were also chosen because the lack of GLR activation leads to reduced cytosolic Ca^2+^ influx, resulting in lower NO production via an l-arginine-dependent and calmodulin-mediated mechanism (Cheng et al. 2018; Adams et al. 2015) (Fig. 1).

### Seed sterilization

For surface sterilization, both wild-type and mutant *Arabidopsis thaliana* seeds were treated with 70% ethanol for 1 min and rinsed five times with sterile deionized water. They were then soaked in 4% sodium hypochlorite solution for 10 min, followed by five washes with sterile deionized water.

### Plant growth and stress treatments

Wild-type and *atglr3.4* mutant *Arabidopsis thaliana* seeds were grown hydroponically in half-strength Hoagland solution (Hoagland and Arnon 1950). Seeds were sown on platforms supporting 54 seeds each and cultivated under a 16/8 h light/dark photoperiod at 25 °C with 60–70% humidity for 21 days. Subsequently, foliar spray treatments were applied (as detailed in Table 1). Harvested plant samples were stored at − 80 °C for further analyses.

**Table 1:** Stress treatments in *A. thaliana* Col-0 and *atglr3.4* mutants grown in a hydroponic system

Applied chemicals	Application method	Application amount	Used ecotypes	Application groups	References
Distilled Water (C)	Spraying method (same duration as other chemicals)	Control	*A. thaliana Col* *atglr3.4.1* *atglr3.4.2*	Col.C *atglr3.4.1* C *atglr3.4.2* C	Li et al. (2014)
Rotenone (R)	Spraying method (10 h)	80 µM	*A. thaliana Col* *atglr3.4.1* *atglr3.4.2*	Col. R *atglr3.4.1* R *atglr3.4.2* R	Li et al. (2014)

C: Control; R: Rotenone

### NO-related methods

#### NO content

NO content was measured according to Zhou et al. (2005). Leaf samples (0.6 g) were homogenized in 3 mL of 50 mM cold acetic acid containing 4% zinc diacetate (pH 3.6). Homogenates were centrifuged at 10,000*g* for 15 min at 4 °C. The supernatants were collected, washed with extraction buffer, and centrifuged again. Charcoal (0.1 g) was added to the supernatants, followed by vortexing and filtration. Griess reagent (1 mL) was then added, and the mixture was incubated at room temperature for 30 min. Absorbance was measured at 540 nm.

#### NOS enzyme activity

NOS activity was determined based on Xiong et al. (2009). NOS activity was measured using the Sigma Nitric Oxide Synthase Detection Kit. The kit uses 4,5-diaminofluorescein (DAF-2DA), which is converted to fluorescent triazofluorescein upon reaction with NO. Fluorescence was measured at excitation and emission wavelengths of 450–495 nm and 505–550 nm, respectively.

#### SNO content

SNO content was determined using the Saville-Griess assay, as described by Lin et al. (2012). Leaf samples were homogenized in extraction buffer containing 50 mM Tris–HCl (pH 8), 1 mM phenylmethanesulfonyl fluoride, and 150 mM NaCl. After incubation on ice, samples were centrifuged at 10,000 rpm. The supernatants were then incubated with sulphanilamide and ethylenediamine in the dark. Absorbance was measured at 540 nm.

#### GSNOR enzyme activity

GSNOR activity was measured by homogenizing leaf samples in a buffer containing 20 mM Tris–HCl (pH 7.5), 2 mM dithiothreitol, 0.2 mM NADH, 0.5 mM EDTA, 0.2% Triton X-100, and 10% glycerol. Homogenates were centrifuged at 27,000*g* for 25 min at 4 °C. The reaction was initiated by adding 0.4 mM GSNO, and GSNOR activity was determined by monitoring NADH oxidation at 340 nm, expressed as nmol NADH consumed per minute per mg of protein (Barroso et al. 2006).

### Methods for determination of ROS

#### H_2_O_2_ content

H_2_O_2_ content was measured according to Cheeseman (2006) with some modifications. The assay used a solution containing 100 μM sorbitol, 250 μM ferrous ammonium sulfate, and 100 μM xylenol orange dissolved in 25 mM H_2_SO_4_. In this modified method, 1% ethanol (EtOH) was added to the ferrous ammonium sulfate/xylenol orange (FOX) buffer. Leaf samples were preserved in liquid nitrogen and homogenized using cold acetone (− 20 °C) with 25 mM H_2_SO_4_. Homogenates were centrifuged at 4000*g* for 5 min at 4 °C. Then, 1 mL of eFOX buffer was added to 50 μL of the supernatant. After 30 min of incubation, absorbance was measured at 550 and 800 nm, and the difference was calculated.

#### O^2⋅−^ content

Superoxide anion (O^2⋅−^) content was measured according to Oracz et al. (2007). Plant samples (0.2 g) were homogenized with 4 mL of 50 mM Na-P buffer (pH 7.8) at 4 °C, followed by centrifugation at 16.000*g* for 15 min. The supernatant (1 mL) was incubated at 25 °C for 30 min in 50 mM Na-P buffer containing 1 mM hydroxylamine. The reaction mixture (0.5 mL) was then incubated with 0.5 mL of 7 mM 2-naphthylamine and 0.5 mL of 17 mM sulphanilamide at 25 °C for another 30 min. After incubation and centrifugation at 13.000*g* for 10 min, absorbance was measured at 540 nm. Superoxide anion content was calculated using a calibration curve generated with sodium nitrite.

### Antioxidant enzyme extraction and activity assays

To minimize protease activity, all analyses were conducted at + 4 °C. Plant samples (0.1 g) were homogenized in 0.5 mL extraction buffer containing 50 mM Tris–HCl (pH 7.8), 0.1 mM EDTA, 0.2% (*v*/*v*) Triton-X100, 1 mM phenylmethylsulfonyl fluoride (PMSF), and 2% (*w*/*v*) polyvinylpolypyrrolidone (PVPP). For ascorbate peroxidase (APX) activity analysis, 5 mM ascorbic acid was also added to the buffer. Homogenates were centrifuged at 14.000 g for 10 min, and supernatants were used for protein quantification and enzyme activity assays. Protein content was determined using the Bradford method (Bradford 1976), with bovine serum albumin (BSA) as the standard.

#### NOX activity

NADPH oxidase activity was measured according to Jiang and Zhang (2002) using a reaction mixture containing 100 μM NADPH and 0.5 mM XTT in 50 mM Tris–HCl buffer (pH 7.5). XTT reduction was monitored at 470 nm. Superoxide dismutase (SOD, 50 U) was used to assess non-enzymatic color changes. O^2⋅−^ production was calculated using an extinction coefficient of 2.16 × 10^4^ M⁻^1^ cm⁻^1^.

#### SOD isozyme and total enzyme activity

Total SOD activity was measured by monitoring the photochemical reduction of nitroblue tetrazolium (NBT) at 560 nm, following the method of Beauchamp and Fridovich (1971). One unit of SOD activity was defined as the amount of enzyme required to inhibit NBT reduction by 50% at 25 °C. SOD isozymes were separated by electrophoresis at + 4 °C on a 12.5% separation gel with a 5% stacking gel. SOD activity was visualized using riboflavin and NBT staining (Beauchamp and Fridovich 1971).

#### CAT activity

Catalase (CAT) activity was measured according to Bergmeyer (1970) by monitoring the decomposition of H_2_O_2_ at 240 nm. One unit of CAT activity was defined as the amount of enzyme that decomposes 1 mmol of H_2_O_2_ per minute.

#### POX activity

Peroxidase (POX) activity was determined according to Herzog and Fahimi (1973). Absorbance was recorded at 465 nm for 3 min, and one unit of POX activity was defined as the amount of enzyme that decomposes 1 mmol of H_2_O_2_ per minute.

#### APX activity

Ascorbate peroxidase (APX) activity was measured according to Nakano and Asada (1981) by monitoring the decrease in absorbance at 290 nm due to ascorbate oxidation. The amount of oxidized ascorbate was calculated using an extinction coefficient of 2.8 mM⁻^1^ cm⁻^1^. One unit of APX activity was defined as the amount of enzyme that oxidizes 1 mmol of ascorbate per minute.

#### GR activity

Glutathione reductase (GR) activity was measured according to Foyer and Halliwell (1976) by monitoring NADPH oxidation at 340 nm. The amount of oxidized NADPH was calculated using an extinction coefficient of 6.2 mM⁻^1^ cm⁻^1^. One unit of GR activity was defined as the amount of enzyme that oxidizes 1 mmol of NADPH per minute.

### Redox-related antioxidants activity assays

#### Determination of GSH/GSSG ratio

GSH and GSSG levels were measured according to Griffith (1980). Leaf samples (0.5 g) were homogenized with cold 2.5 M HClO_2_, centrifuged at 16,000*g* for 10 min, and the resulting supernatant was mixed with DTNB and GR. Absorbance changes at 412 nm were used to calculate GSH and GSSG levels.

#### Measurement of ascorbate levels

Ascorbate content was determined using the Fe^3^⁺ to Fe^2^⁺ reduction method by Law et al. (1983). Leaf tissue (0.1 g) was homogenized in 10% TCA, and the supernatant was incubated with NaOH, TCA, Na-phosphate buffer, DTT, H_3_PO_4_, N-ethylmaleimide, and FeCl_3_ at 37 °C for 60 min. Absorbance was measured at 525 nm.

#### GST and GPX activity

GST and GPX activities were determined using methods from Habig et al. (1974). For GST, the reaction mixture contained 0.1 M Na-phosphate buffer (pH 6.5), 1 mM GSH, and 1 mM CDNB. For GPX, the reaction mixture contained 0.2 mM NADPH, 1 mM sodium azide, 1 mM GSH, 1 U GR, and 2 mM H_2_O_2_. NADPH oxidation was measured at 340 nm, with one unit of GPX defined as the amount of enzyme oxidizing 1 mmol of NADPH per min.

#### NAD/NADH ratio

NAD/NADH ratio was measured using the reduction of DCPIP in the presence of ethanol and PMS as described by Queval and Noctor (2007). Absorbance changes were monitored at 600 nm, and NAD^+^ or NADH content was calculated using a standard curve.

#### MDHAR activity

MDHAR activity was measured using the method of Arrigoni et al. (1981), involving a reaction mixture containing K-P buffer, ascorbic acid, NADH, and ascorbate oxidase. NADH oxidation was monitored at 340 nm, and activity was calculated using an extinction coefficient of 6.2 mM⁻^1^ cm⁻^1^.

#### DHAR activity

DHAR activity was measured following Nakano and Asada (1981) using a reaction mixture containing K-P buffer, dehydroascorbate, GSH, and EDTA. Ascorbate formation was measured at 265 nm using an extinction coefficient of 0.98 mM⁻^1^ cm⁻^1^.

### Lipid peroxidation (TBARS)

Lipid peroxidation was assessed by measuring malondialdehyde (MDA) levels using the TBA reaction as described by Madhava Rao and Sresty (2000). Absorbance was measured at 532 nm, and non-specific turbidity at 600 nm was subtracted. MDA concentration was calculated using an extinction coefficient of 155 mM⁻^1^ cm⁻^1^.

### Protein oxidation

Oxidative damage to proteins was assessed by measuring carbonyl group levels using the DNPH reduction method according to Levine et al. (1994). Absorbance was measured at 370 nm.

### Measurement of gene expressions by RT-PCR

Quantitative Real-Time PCR (qRT-PCR) was conducted to compare gene expression levels under various abiotic stress conditions. Total RNA was extracted from plant tissues of both stress and control groups using the Qiagen RNeasy® Plant Mini Kit, following the manufacturer’s protocol. The purity (1.8–2.0) and quantity of the isolated RNA were checked spectrophotometrically at 260/280 nm. After confirming the absence of DNA contamination, cDNA synthesis was carried out using the Transcriptor High Fidelity cDNA Synthesis Kit (Roche) according to the supplier's guidelines. PCR reactions were performed using the ABI Step-One Plus system, and ABI-SYBR Green Master Mix was used for amplification. The expression of each target gene was normalized against the reference gene Actin1 (ACT1), and relative expression levels were calculated. The qPCR analysis was conducted using the Step OnePlus™ Real-Time PCR System (Applied Biosystems), and data were analyzed with the StepOne™/StepOnePlus™ Software v2.3. The relative gene expression levels were calculated using the “comparative Ct” method. Specifically, the “2-ΔΔCq (Livak) Method” was applied to quantify target gene expression relative to the control group.

### Statistical analysis

All experiments were conducted in duplicate with three biological replicates (*n* = 6). Results are presented as the mean ± standard error of the mean. To check for normal distribution, the Shapiro–Wilk test was used, followed by one-way ANOVA. The homogeneity of variances among groups was assessed using Levene's test. Tukey's test was applied for pairwise comparisons when variances were equal, while the Games-Howell test was used if variances were unequal. For gene expression analysis, each genotype's stress group was compared with its corresponding control group.

## Results

### NO related findings

**Fig. 2:**
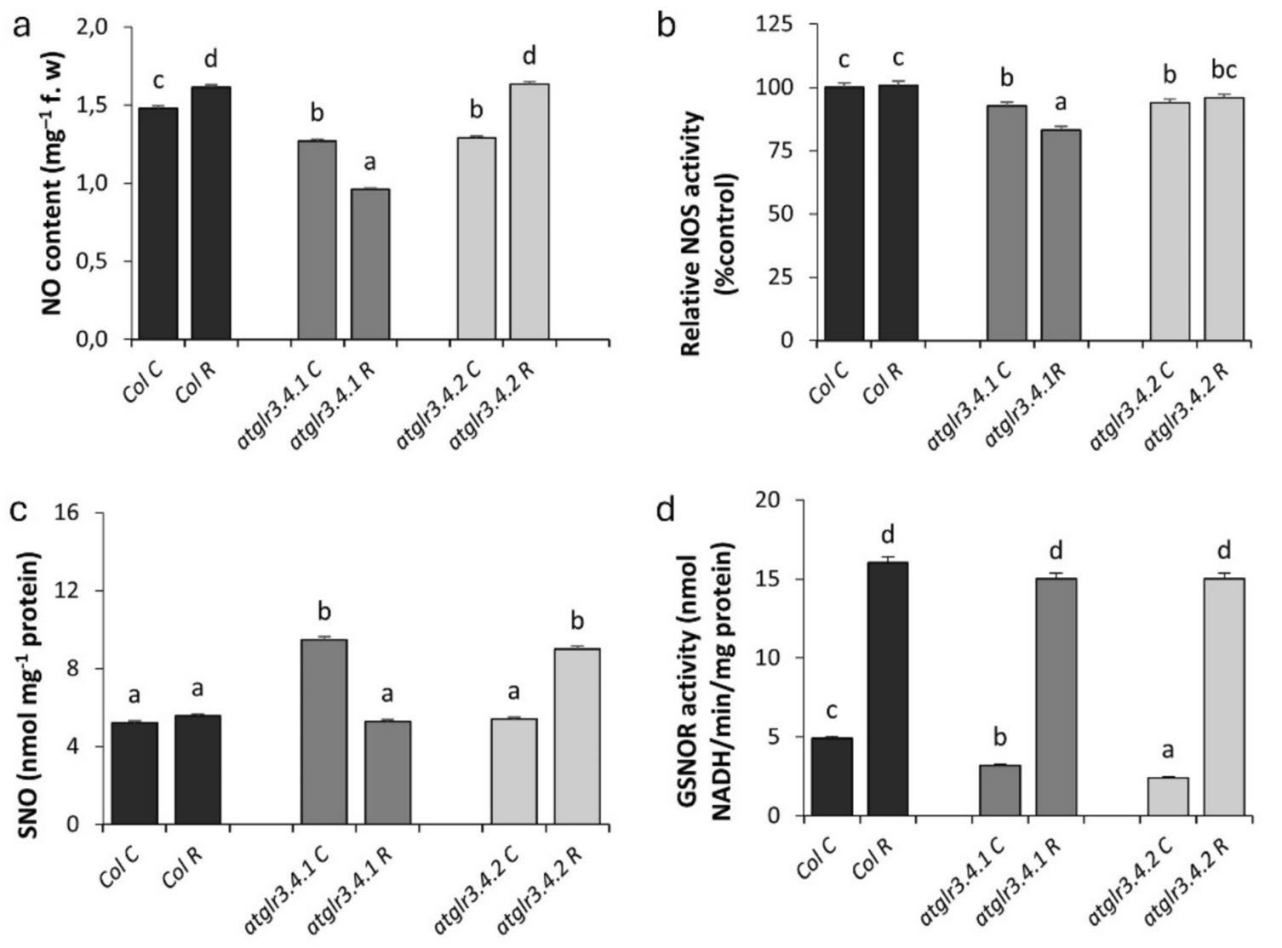
The effects of exogenous Rotenone (R) treatment on NO (**a**), NOS (**b**), SNO (**c**), and GSNOR (**d**) in wild-type *Arabidopsis thaliana* Col. and *atglr3.4* mutants. Letters indicate statistically significant differences (*p* < 0.05)

NO content was determined at AtGLR3.4 receptors. *atglr3.4.1*and *atglr3.4.2* knockout reduced basal NO levels by 14% and 13%, respectively. Rotenone (R) induced a 10% increase in NO in Col., with no additional effect observed in *atglr3.4* knockout mutants (Fig. 2a). Another NO-related analysis performed is NOS activity. Under normal conditions, NOS activity was decreased by 10% in *atglr3.4.1* and *atglr3.4.2* mutants compared to Col. Exogenous R application increased NOS activity of Col. by only 6% compared to control. Indeed, *atglr3.4.1* knockout decreased NOS activity by 20%, while *atglr3.4.2* knockout caused only an 8% decrease for Col (Fig. 2b). The SNO content is, under untreated conditions, *atglr3.4.1* knockout caused a significant increase of 81% in SNO amount, whereas *atglr3.4.2* knockout did not cause any change. Exogenous rotenone application of caused a modest 6% increase in SNO levels in Col., with *atglr3.4.1* knockout having no additional effect, while *atglr3.4.2* knockout resulted in a 61% increase (Fig. 2c). Finally, GSNOR enzyme activity was determined at AtGLR3.4 receptors. Under control conditions, GSNOR activity decreased by 35% in *atglr3.4.1* and 50% in *atglr3.4.2* mutants compared to Col. Rotenone treatment significantly increased GSNOR activity, leading to a 3.2-fold increase in Col. compared to the control. This rotenone-induced increase was reduced by approximately 6% in both *atglr3.4.1* and *atglr3.4.2* knockout plants (Fig. 2d).

### Determination of ROS

**Fig. 3:**
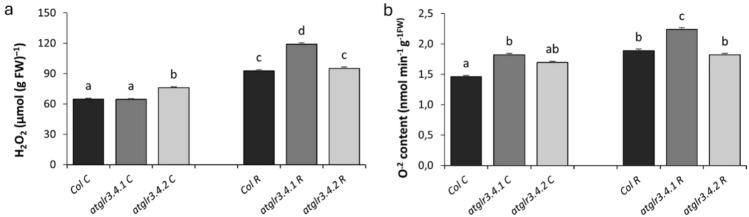
The effects of exogenous Rotenone (R) treatment on H_2_O_2_ (**a**) and O^2⋅−^ (**b**) contents in wild-type *Arabidopsis thaliana* Col. and *atglr3.4* mutants. Letters indicate statistically significant differences (*p* < 0.05)

In the study, H₂O₂ and O^2⋅−^ were determined from ROS. Under normal conditions, *atglr3.4.1* knockout had no effect on H₂O₂ levels, whereas *atglr3.4.2* knockout led to a 17% increase. Rotenone treatment elevated H₂O₂ levels in Col. by 43%, with *atglr3.4.1* knockout further increasing it by 28%, whereas *atglr3.4.2* knockout had no significant effect (Fig. 3a). In the untreated conditions, the knockout of AtGLR3.4.1 and AtGLR3.4.2 genes caused an increase of 25% and 16% in O^2⋅−^ amount, respectively. Exogenous Rotenone (R) application increased the O^2⋅−^ content of Col. by 30% compared to the control. However, *atglr3.4.1* knockout increased the O^2⋅−^ amount by 22%, while *atglr3.4.2* knockout did not change the O^2⋅−^ content (Fig. 3b).

### Antioxidant enzyme activity

NADPH oxidase (NOX), superoxide dismutase (SOD), catalase (CAT), peroxidase (POX), ascorbate peroxidase (APX) and glutathione reductase (GR) enzymes activity were identified in our study.

**Fig. 4:**
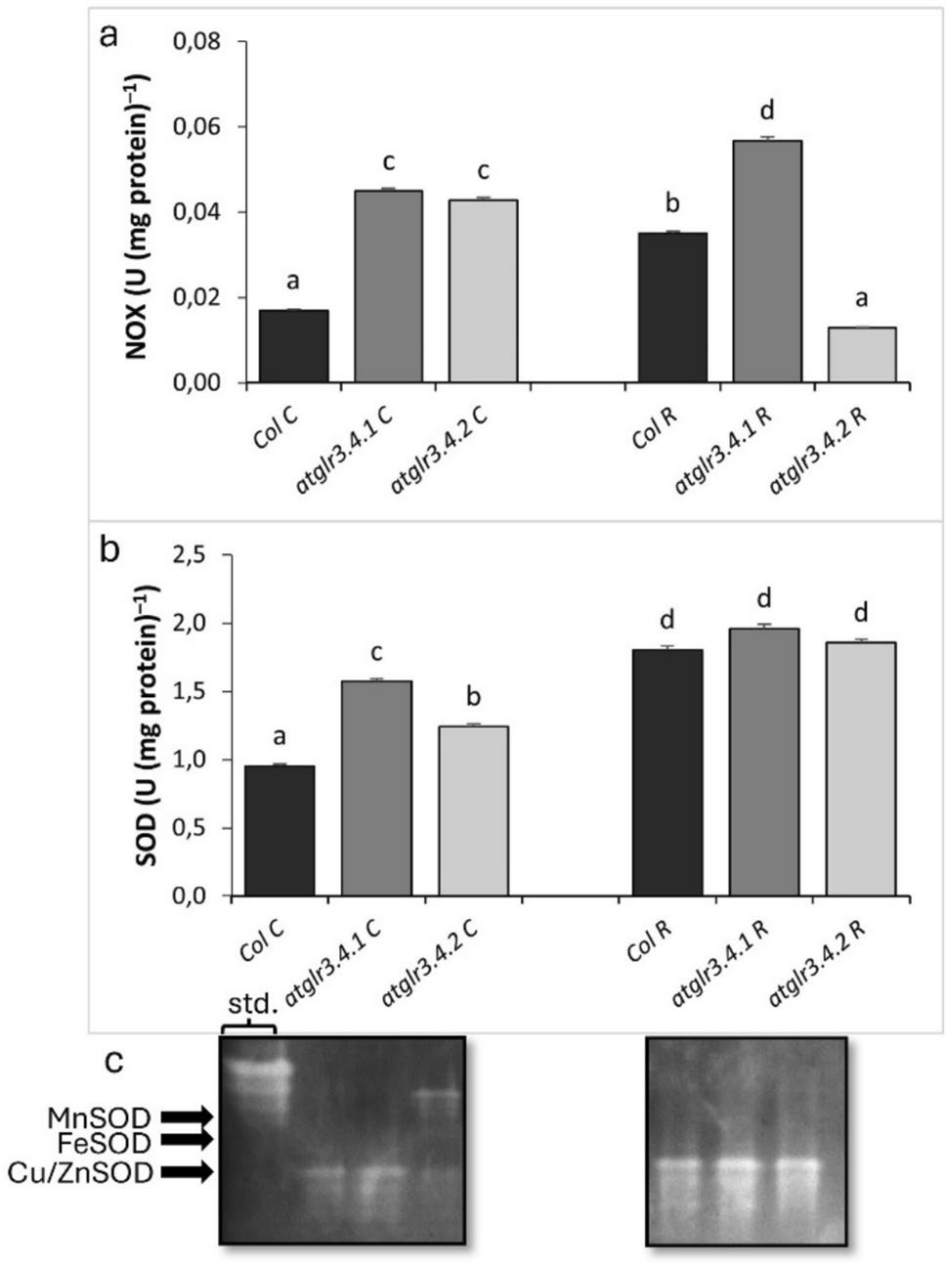
The effects of exogenous Rotenone (R) treatment on NOX (**a**) SOD (**b**), and isoenzyme patterns (**c**) activities in wild-type *Arabidopsis thaliana* Col. and *atglr3.4* mutants. Letters indicate statistically significant differences (*p* < 0.05)

**Fig. 5:**
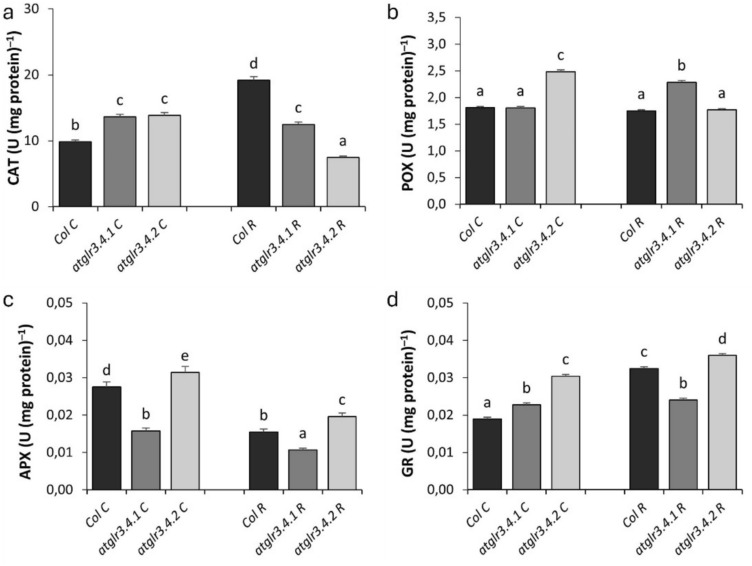
The effects of exogenous Rotenone (R) treatment on CAT (**a**), POX (**b**), APX (**c**) and GR (**d**) activities in wild-type *Arabidopsis thaliana* Col. and *atglr3.4* mutants. Letters indicate statistically significant differences (*p* < 0.05)

NOX activity, in control conditions, *atglr3.4.1* and *atglr3.4.2* knockouts caused an approximately 2.5-fold increase. Exogenous Rotenone application increased Col.’s NOX activity approximately twofold compared to the control (Fig. 4a). In *atglr3.4.1* knockout, a 62% increase was observed in NOX activity over Col. R, while a 63% decrease was observed in *atglr3.4.2* knockout. SOD Activity under control conditions, GLR3.4.1 knockout increased SOD activity by 64%, while *atglr3.4.2* knockout led to a 30% increase (Fig. 4b). Cu/ZnSOD isozyme activity was more pronounced in *atglr3.4.1* knockout plants, whereas MnSOD and FeSOD isozymes were more prominent in *atglr3.4.2* knockout plants. Rotenone treatment increased SOD activity in Col. by 88% compared to the control, but this increase was not observed in *atglr3.4.1* and *atglr3.4.2* knockout plants. Only Cu/ZnSOD isozyme activity was detected across all genotypes under rotenone (Fig. 4c). Under normal conditions CAT activity, *atglr3.4.1* and *atglr3.4.2* knockout increased CAT activity by 38% and 40%, respectively. Rotenone application doubled CAT activity in Col. relative to the control (Fig. 5a). However, *atglr3.4.1* and *atglr3.4.2* knockout decreased this activity by 34% and 61%, respectively. When it comes to POX activity, under normal conditions, *atglr3.4.1* knockout did not significantly affect POX activity, whereas *atglr3.4.2* knockout increased it by 37%. Rotenone application did not alter POX activity in Col. However, *atglr3.4.1* knockout increased POX activity by 30%, while *atglr3.4.2* knockout had no effect (Fig. 5b). APX activity was decreased by 40% under control conditions with *atglr3.4.1* knockout, while AtGLR3.4.2 gene knockout increased it by 14%. Exogenous R application caused a 43% decrease in APX activity of Col. compared to control. Similarly, *atglr3.4.1* knockout further reduced this decrease by 31%. However, AtGLR3.4.2 gene knockout increased APX activity by 27% (Fig. 5c). Under normal conditions GR activity, AtGLR3.4.1 and AtGLR3.4.2 gene knockout increased by 20% and 60%, respectively. Rotenone application caused a 72% increase in GR activity in Col. compared to the control, with a 26% reduction in this increase due to *atglr3.4.1* knockout, while *atglr3.4.2* knockout further increased GR activity by 10% (Fig. 5d).

### Redox related antioxidant activity analyses

In our study, total glutathione ratio (GSH/GSSG), ascorbate content, glutathione-S transferase (GST), glutathione peroxidase (GPX), NAD/NADH ratio, monodehydroxyascorbate reductase (MDHAR) and dehydroxyascorbate reductase (DHAR) was determined.

**Fig. 6:**
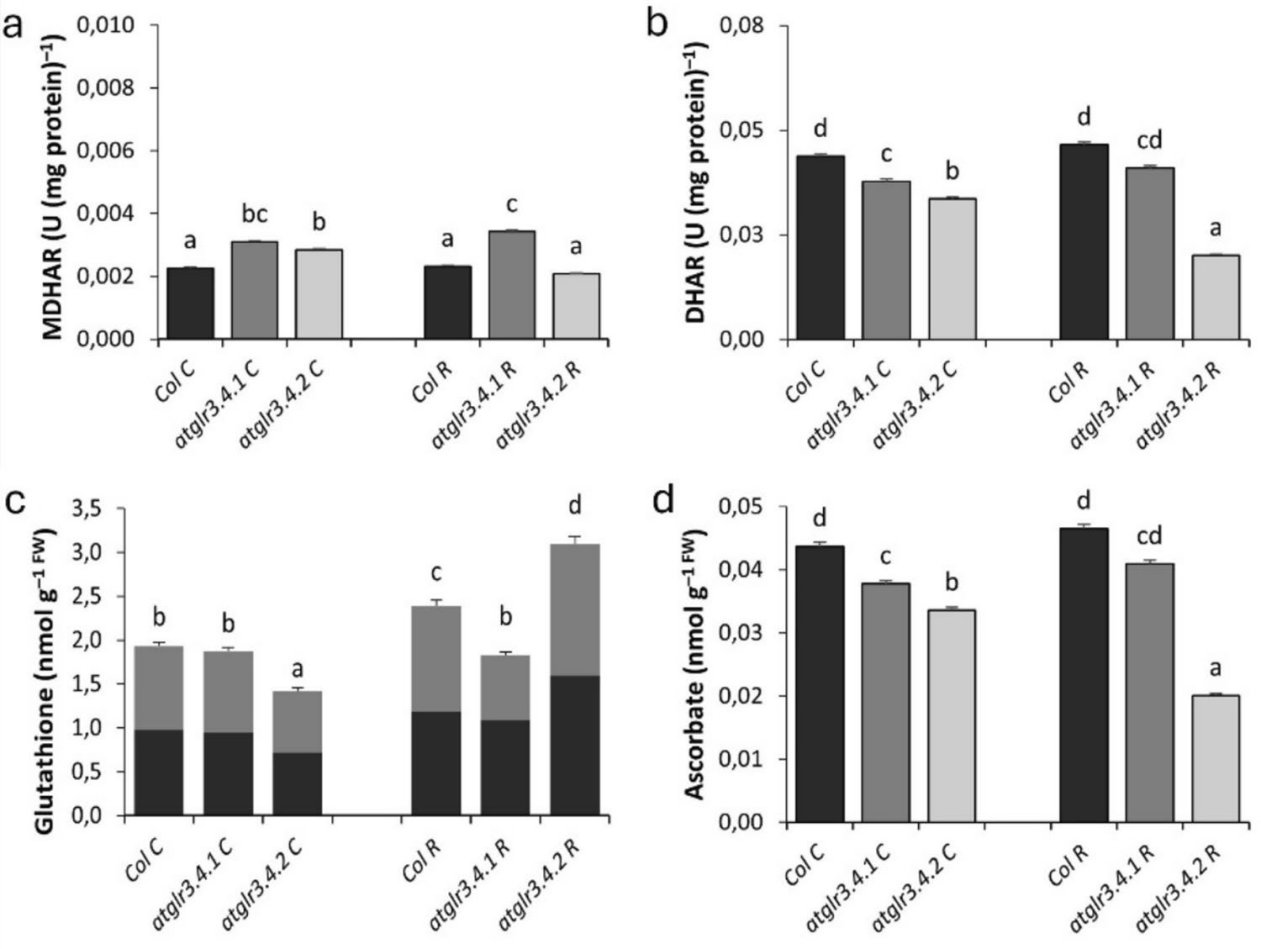
The effects of exogenous Rotenone (R) treatment on MDHAR (**a**) and DHAR (**b**) activities, (**c**) Total Glutathione and Ascorbate (**d**) Content in wild-type *Arabidopsis thaliana* Col. and *atglr3.4* mutants. Letters indicate statistically significant differences (*p* < 0.05)

**Fig. 7:**
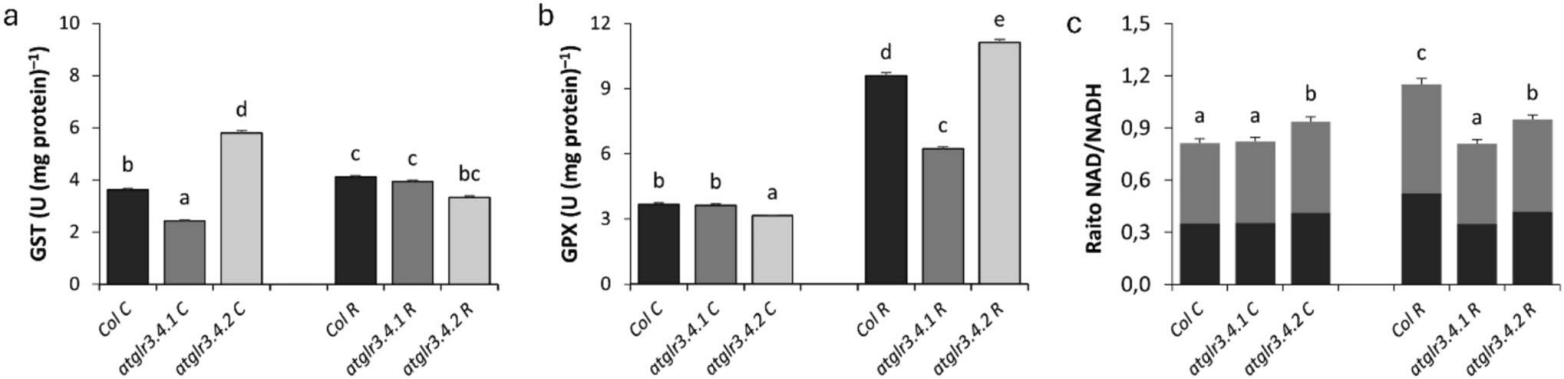
The effects of exogenous Rotenone (R) treatment on GST (**a**), GPX (**b**) and NAD/NADH (**c**) in wild-type *Arabidopsis thaliana* Col. and *atglr3.4* mutants. Letters indicate statistically significant differences (*p* < 0.05)

GSH/GSSG Ratio under control conditions, AtGLR3.4.1 gene knockout did not change total glutathione content, while *atglr3.4.2* knockout decreased it by 26%. Rotenone application increased glutathione content in Col. by 24%. *atglr3.4.1* knockout did not affect this increase, while *atglr3.4.2* knockout caused a 30% increase (Fig. 6c). Ascorbate content decreased in both *atglr3.4.1* and *atglr3.4.2* knockout, by 13% and 23%, respectively. Rotenone increased ascorbate content by 6%, which decreased by 12% with GLR 3.4.1 knockout and by 56% with *atglr3.4.2* knockout (Fig. 6d). *atglr3.4.1* knockout decreased GST activity by 33%, while *atglr3.4.2* knockout increased it by 60%. Rotenone increased GST activity by 13%, but *atglr3.4.1* knockout did not affect this increase, while *atglr3.4.2* knockout reduced GST activity by 18% (Fig. 7a). *atglr3.4.1* knockout did not affect GPX activity under control conditions, while *atglr3.4.2* knockout decreased it by 14%. Rotenone application increased GPX activity by 2.6-fold, but this increase was reduced by 35% in *atglr3.4.1* knockout, while it increased by 15% in *atglr3.4.2* knockout (Fig. 7b). Under normal conditions NAD/NADH ratio did not change with AtGLR3.4.1 gene knockout, but this ratio increased by 14% with AtGLR3.4.2 gene knockout. Exogenous Rotenone application increased Col.’s NAD/NADH ratio by 41% compared to control. However, this rise decreased by 29% with *atglr3.4.1* knockout, while a 17% decrease was observed in NAD/NADH ratio with *atglr3.4.2* knockout (Fig. 7c). Under untreated conditions, *atglr3.4.1* knockout increased MDHAR activity by 36%, while *atglr3.4.2* knockout increased it by 25%. Exogenous Rotenone did not affect MDHAR activity in Col., but *atglr3.4.1* knockout increased it by 47%, while *atglr3.4.2* knockout decreased it by 10% (Fig. 6a). Under normal conditions, *atglr3.4.1* knockout reduced DHAR activity by 13%, and *atglr3.4.2* knockout reduced it by 23%. Rotenone application increased DHAR activity in Col. by 6%, with *atglr3.4.1* knockout further reducing it by 12%, and *atglr3.4.2* knockout decreasing it by 56% (Fig. 6b).

### Lipid peroxidation (TBARS) and protein oxidation

**Fig. 8:**
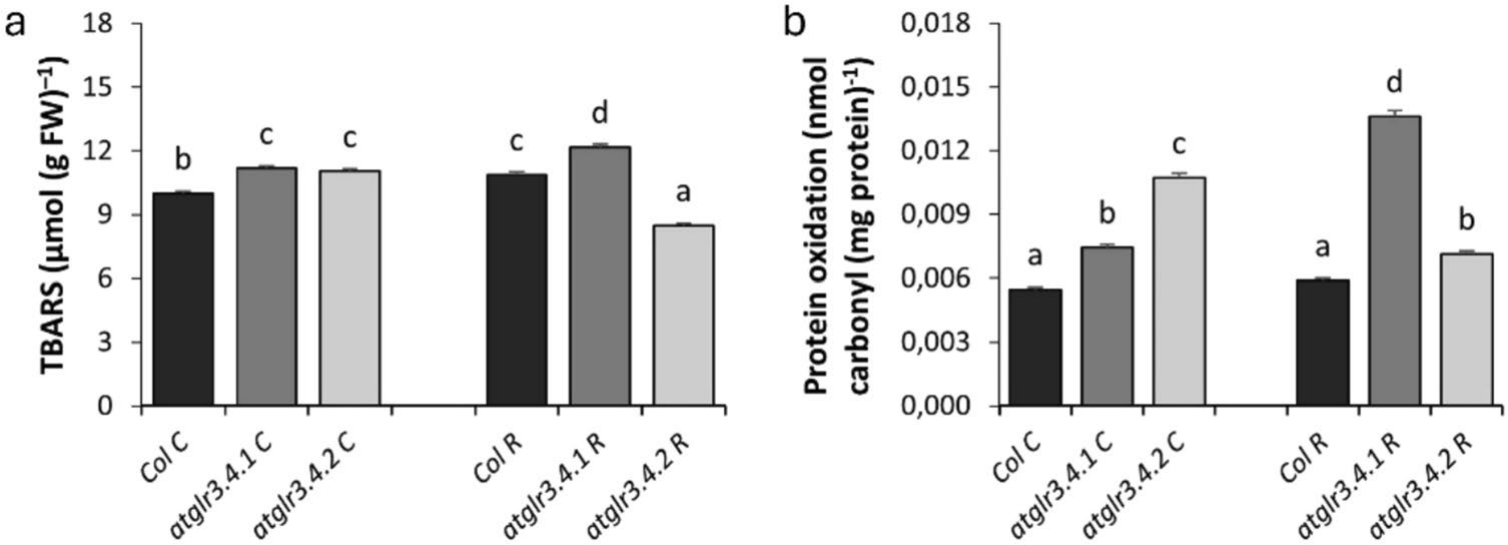
The effects of exogenous Rotenone (R) treatment on TBARS (**a**) and Protein Oxidation (**b**) in wild-type *Arabidopsis thaliana* Col. and *atglr3.4* mutants. Letters indicate statistically significant differences (*p* < 0.05)

Under controlled conditions, AtGLR3.4.1 and AtGLR3.4.2 gene knockout caused a 10% increase in lipid peroxidation. Rotenone application increased the TBARS amount of Col. by 11% compared to the control. However, AtGLR3.4.1 gene knockout increased the TBARS amount by 12%, while AtGLR3.4.2 gene knockout decreased it by 22% (Fig. 8a).

Protein oxidation caused AtGLR3.4 gene knockout even under normal conditions and increased by 35% and 95% in AtGLR3.4.1 and AtGLR3.4.2 gene knockout, respectively, compared to Col. control. Exogenous Rotenone application increased protein oxidation of Col. by only 7% compared to control. This rise was increased by 1.5-fold with *atglr3.4.1* knockout and by 21% with *atglr3.4.2* knockout (Fig. 8b).

### Determination of mitochondrial stress-related gene expressions

Complex I (CI7B: 76 kDa subunit of complex I), upregulated by oxidative stress (UPOX), alternative oxidase (AOX1), uncoupling protein (UCP), succinate dehydrogenase 2–1 in complex II (SDH2E) and 5b subunit of cytochrome c oxidase (complex IV) (COX5b) genes were determined in our study.

**Fig. 9:**
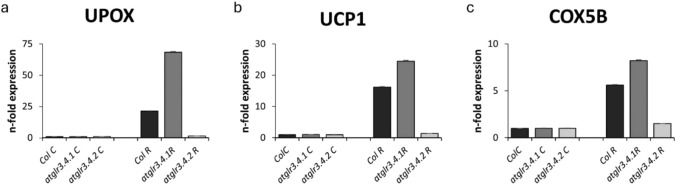
The effect of exogenous Rotenone (R) treatment on CI7B (**a**), AOX1 (**b**) and SDH2E (**c**) transcriptions in wild-type *Arabidopsis thaliana* Col. and *atglr3.4* mutants. Different letters indicate statistically significant differences (*p* < 0.05)

**Fig. 10:**
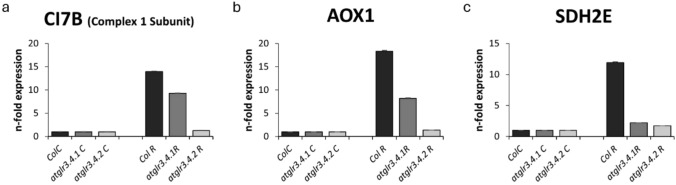
The effect of exogenous Rotenone (R) treatment on UPOX (**a**), UCP1 (**b**) and COX5B (**c**) transcriptions in wild-type *Arabidopsis thaliana* Col. and *atglr3.4* mutants. Different letters indicate statistically significant differences (*p* < 0.05)

The conditions applied with rotenone caused a 14-fold increase in the transcription of Col. Complex I (CI7B: 76 kDa subunit of complex I) compared to the control. This increase was reduced by the knockout of AtGLR3.4.1 compared to the control but was reduced by the knockout of AtGLR3.4.2 gene and brought it to the control level (Fig. 10a). In UPOX transcription level, rotenone application caused a 21-fold increase in Col. compared to the control, while AtGLR3.4.1 gene knockout increased by approximately threefold compared to Col. (Fig. 9a). AOX1 transcription was the greatest increase in Col. with Rotenone application compared to control. This increase was 18.5-fold. The knockout of AtGLR3.4.1 gene decreased this increase approximately 2.5-fold, however, knockout of AtGLR3.4.2 decreased AOX1 transcription and brought it to control level (Fig. 10b). Rotenone application increased UCP1 transcription in Col. approximately 14-fold compared to control, while *atglr3.4.1* knockout caused a 2.6-fold to enhance in this increase, *atglr3.4.2* knockout decreased this increase and brought it to control level (Fig. 9b). R treatment caused a 12-fold increase in SDH2E transcription of Col. compared to control. However, with the AtGLR3.4.1 gene knockout, this enhance decreased approximately fivefold, while with the AtGLR3.4.2 gene knockout, SDH2E transcription decreased and reached control level (Fig. 10c). COX5B transcription of Col., under rotenone application caused a sixfold increase in compared to control, while *atglr3.4.1* knockout increased it by eightfold compared to control. However, the knockout of AtGLR3.4.2 gene did not change compared to control (Fig. 9a).

## Discussion

*Arabidopsis thaliana* is a model organism for plant biology and an important tool for understanding the molecular mechanisms of human diseases such as neurodegenerative diseases (Xu and Moller 2011). Among other known pathways, in humans, NMDAR-mediated NO accumulation leads to inhibition of the PDI enzyme, causing mitochondrial stress and predisposing to Alzheimer's and Parkinson's disease (Uehara et al. 2006). It is not known whether these pathways are similar in plants. However, it has been shown that NMDAR-like GLR receptors are also present in Arabidopsis and that lack of GLR3.4 activation reduces the Ca^2^⁺ level in the cytosol (Lam et al. 1998; Cheng et al. 2018). This study aims to examine how knockout of GLR3.4.1 and GLR3.4.2 affects ROS-RNS balance under rotenone-induced mitochondrial stress and its impact on antioxidant defense systems.

In our study, it was determined that knockout of GLR3.4.1 and GLR3.4.2 genes caused a significant decrease in NO levels. When mitochondrial stress (induced by Rotenone) was applied, NO content increased in the Col line. Interestingly, NO levels in the GLR3.4.2 knockout under this stress were like those in Col. On the contrary, it caused a significant decrease in NO amount in GLR3.4.1 knockout. This suggests that GLR3.4.2 genes may not play a direct role in regulating NO levels during mitochondrial stress responses, or that other mechanisms might be controlling these responses. Additionally, it is plausible that GLR3.4.1 is involved in a mechanism that normally promotes NO production, and its loss may have resulted in a reduction in NO synthesis. This suggests that GLR3.4.2 is not the primary regulator of these pathways, while GLR3.4.1 is likely to be involved in a mechanism that promotes NO production under mitochondrial stress (Fig. 2a).

In plant systems, NO production occurs via nitrate-dependent and L-arginine-dependent pathways. In mammals, NOS enzymes catalyze NO production from L-arginine (Domingos et al. 2015). Although there is limited evidence for the presence of NOS in plants, the presence of the AtNOS1 gene in *Arabidopsis thaliana* has been shown to be like mammals (Guo et al. 2003; Crawford et al. 2006).

Under control conditions, both NO levels and NOS activity in the mutants were found to be lower compared to the wild-type Col. This suggests that the absence of GLR3.4.1 and GLR3.4.2 genes may directly affect both NO production and NOS activity. Under mitochondrial stress, NO levels increased in all genotypes compared to their respective controls. Interestingly, the NO levels were equal among the three genotypes. However, while NOS activity increased in parallel with NO levels in Col and the *atglr3.4.2* mutant, it decreased in the *atglr3.4.1* mutant. This observation suggests that the NO production pathway is activated under mitochondrial stress, but NOS activity is differentially regulated among the mutants (Fig. 2b).

Unlike other genotypes, the decrease in NOS activity observed in the *atglr3.4.1* mutant under mitochondrial stress may be due to the inhibitory effect of rotenone on Complex I. Previous studies have shown that mtNOS is functionally related to Complex I, and when Complex I is activated, mtNOS exhibits high activity (Parihar et al. 2008). In this context, the decrease in NOS activity under mitochondrial stress may be attributed to the inhibition of Complex I by rotenone. However, despite this decrease in NOS activity, the increase in NO content observed in the *atglr3.4.1* mutant suggests that an alternative NO synthesis pathway may be activated. This could be explained by the involvement of the nitrate reductase (NR) pathway. In this context, the observed increase in NOS activity alongside NO accumulation in Col and *atglr3.4.2* mutants support the role of NOS in regulating cellular NO levels. However, the decrease in NOS activity in the *atglr3.4.1* mutant suggests that different regulatory mechanisms may be involved in this genotype. This finding indicates that GLR3.4.1 and GLR3.4.2 genes may have distinct roles in regulating NO production under mitochondrial stress conditions. Specifically, different GLR isoforms may influence NOS activity and consequently NO production in response to stress in distinct ways (Fig. 2b).

S-Nitrosoglutathione (GSNO), as the storage and transport form of NO, transfers NO signaling to other proteins via trans-nitrosylation and thus plays a critical role in NO signaling. GSNO and S-nitrosylated protein levels are regulated by GSNO-reductase (GSNOR). GSNOR is a conserved enzyme that protects cells under nitrosative stress and regulates NO signaling by balancing S-nitrosothiol (SNO) levels (Corpas et al. 2011; Lindermayr 2018). Despite the short half-life of NO, SNOs are more stable and play a role in the transport, storage and post-translational modifications of NO (Lindermayr and Durner 2009). In our study, GLR3.4.2 knockout did not affect the amount of SNO, while GLR3.4.1 knockout increased the level of SNO approximately two-fold; under the same conditions, GSNOR activity did not change in GLR3.4.2 knockout, but decreased in GLR3.4.1 knockout (Fig. 2c).

Previous studies have shown that intracellular SNO content is inversely proportional to GSNOR activity (Rusterucci et al. 2007; Lee et al. 2008). In this regard, it can be stated that the inverse relationship between GSNOR activity and SNO levels is crucial for maintaining the NO balance in GLR mutants. Positive correlation was observed between GSNOR and SNO by Frungillo et al. (2013) in *A. thaliana* under nutrient stress. As it is known, GSNOR activity plays an important role in maintaining cellular redox balance by interacting with ROS (Jahnova et al. 2019). Under control conditions, a decrease in GSNOR activity in both mutants suggests that these two genes may play a role in cellular redox homeostasis (Fig. 2d). However, despite the accumulation of S-nitrosothiols (SNO) in the GLR3.4.1 knockout, SNO levels do not change in the GLR3.4.2 knockout, indicating that the effects of these two genes on GSNOR might occur through different mechanisms. The reduction in GSNOR activity in the GLR3.4.2 knockout, coupled with unchanged SNO levels, suggests that GLR3.4.2 could regulate SNO homeostasis through a mechanism independent of GSNOR. In response to rotenone-induced mitochondrial stress, the increase in GSNOR activity in the *atglr3.4.1* mutant indicates an attempt by the cell to maintain the balance of nitric oxide (NO) and SNO. However, the decreased SNO levels observed in this mutant despite the increase in GSNOR activity implies that the overactivation of GSNOR may have led to enhanced SNO degradation. Additionally, it is plausible that GLR3.4.1 is involved in a mechanism that normally promotes NO production, and its loss may have resulted in a reduction in NO synthesis. This hypothesis is supported by the observed significant reduction in NO levels in the *atglr3.4.1* mutant under mitochondrial stress. On the other hand, the lack of GLR3.4.2 might disrupt SNO homeostasis, potentially leading to an excessive accumulation of SNO. Although the cell attempts to regulate this increase by upregulating GSNOR activity, other factors could interfere with the efficient functioning of GSNOR. For instance, the increased levels of hydrogen peroxide (H₂O₂) under stress conditions in the *atglr3.4.2* mutant may have contributed to the inhibition of GSNOR. Taken together, these results suggest that GLR3.4.1 and GLR3.4.2 play distinct roles in NO metabolism. GLR3.4.1 appears to primarily promote SNO production, while GLR3.4.2 functions to suppress SNO accumulation. These findings underscore the differential roles of these genes in regulating the redox state and stress responses within the cell (Fig. 2c, d).

AtGLR3.4 glutamate receptor-like channels are notable for their Ca^2^⁺ permeability, and Ca^2^⁺ plays a significant secondary messenger role in regulating cellular functions (Vincill et al. 2012; Berridge 2012). Ca^2^⁺ interacts with reactive oxygen species (ROS), and changes in intracellular Ca^2^⁺ levels can affect ROS production and signaling (Görlach et al. 2015). Additionally, nitric oxide (NO) production is known to be necessary for H₂O₂ production, and H₂O₂ promotes NO synthesis during stomatal closure (Lu et al. 2009). In our study, a significant increase in H₂O₂ and superoxide (O^2⋅−^) levels was observed in the *atglr3.4.1* and *atglr3.4.2* mutants compared to Col., and this increase was determined to be associated with NADPH oxidase (NOX) activity (Fig. 4a). Notably, all genotypes increased H₂O₂ more under mitochondrial stress conditions. The highest increase was observed in the *atglr3.4.1* mutant under mitochondrial stress. Similarly, the amount of O^2⋅−^ radicals increased in all genotypes under mitochondrial stress. However, the *atglr3.4.1* mutant exhibited the highest levels of both H₂O₂ and O^2⋅−^ radicals compared to other genotypes (Fig. 3a, b). These findings provide valuable insights into the potential effects of mitochondrial stress on ROS production and the roles of GLR genes in this process.

In our study, the NADH dehydrogenase complex (Complex I), a component of the electron transport chain (ETS), was inhibited using rotenone (Newhouse et al. 2004; Radad et al. 2006). Complex I is a major source of ROS production in mitochondria, and modulation of its activity can directly influence the organism's lifespan. Under normal conditions, electrons from Complex I are transferred to Complex III via Coenzyme Q. However, under certain conditions, electrons can be transferred back from Ubiquinol to Complex I, a phenomenon known as Reverse Electron Transport (RET), which leads to ROS production. Rotenone inactivates Complex I, promoting RET and subsequently increasing intracellular ROS levels. This explains the increase in radical levels observed in all genotypes treated with rotenone. However, the more pronounced radical accumulation observed in the *atglr3.4.1* mutant compared to *atglr3.4.2* mutants suggests that this mutant might be more sensitive to rotenone inactivating Complex I (Fig. 10a).

The greater sensitivity of the *atglr3.4.1* mutant to Complex I inhibition compared to the *atglr3.4.2* mutant suggests that GLR3.4.1 may serve as a key regulator of the oxidative stress response. Complex I inhibition not only enhances ROS production via reverse electron transport (RET) but also upregulates the expression of mitochondrial stress-related genes. The fact that these effects are more pronounced in the *atglr3.4.1* mutant indicates that the loss of GLR3.4.1 disrupts mitochondrial redox homeostasis, increases ROS levels, and excessively activates the mitochondrial stress response. These findings suggest that, compared to GLR3.4.2, GLR3.4.1 may play a more protective role in maintaining mitochondrial function and regulating oxidative stress responses. GLR3.4.2 appears to have a role in regulating ROS accumulation (Fig. 3a, b).

Accumulation of reactive oxygen species (ROS) triggered by various stressors is mitigated by enzymatic antioxidants like SOD, POX, APX, GR, and CAT, as well as non-enzymatic metabolites such as ascorbate and glutathione, helping to maintain cellular redox homeostasis (Mittler et al. 2004; Gill et al. 2011). SOD converts O^2⋅−^ into H₂O₂, which is then detoxified by CAT (Mittler 2011). POX facilitates redox reactions involving H₂O₂, while APX acts as a primary H₂O₂ scavenger in chloroplasts and the cytosol (Asada 2006). In this study, the loss of GLR3.4.1 and GLR3.4.2 led to elevated NOX activity under normal conditions, likely to compensate for reduced cytoplasmic Ca^2^⁺ by activating Ca^2^⁺ channels (Foreman et al. 2003). This increase in NOX activity elevated O^2⋅−^ levels, consequently boosting SOD activity, with the highest activity observed in *atglr3.4.1* and *atglr3.4.2* mutants (Fig. 4a, b). This might be linked to post-translational modifications such as S-nitrosylation, as these mutants exhibited higher SNO levels (Fig. 2c). On the other hand, rotenone-induced mitochondrial stress due to the inhibition of Complex I and increased NOX activity led to significant accumulation of O^2⋅−^ and H₂O₂ in all genotypes. As a result, the enzyme responsible for scavenging O^2⋅−^, SOD, was upregulated in all genotypes. To prevent the excessive accumulation of H₂O₂ produced by this reaction, CAT served as the primary scavenger enzyme in the Col. genotype. In contrast, in the *atglr3.4.1* mutant, POX took on this role, while in the *atglr3.4.2* mutant, APX assumed this function. Interestingly, in both mutants, the activity of CAT, one of the H₂O₂ scavengers, was decreased (Fig. 5a, b).

Glutathione is an important tripeptide found in various cellular compartments and plays a critical role in maintaining cellular redox balance, the regeneration of reduced AsA, combating oxidative stress, particularly through the regulation of the GSSG/GSH ratio (Shao et al. 2008; Halliwell and Foyer 1976) and the functionality of enzymes such as DHAR, GR, GST, and GPX that use glutathione as a substrate (Jimenez et al. 1997). The GSH/GSSG ratio is crucial for maintaining redox homeostasis during H₂O₂ detoxification (Shao et al. 2008). In our study, both Col. and *atglr3.4.2* mutants increased total glutathione levels to prevent GSH depletion during rotenone-induced mitochondrial stress, which was accompanied by an increase in GR and GPX activities (Figs. 5d, 7a). However, in *atglr3.4.2* mutants, GR, GPX, and DHAR activities were reduced, while ascorbate levels decreased, impairing glutathione pool replenishment and disrupting cellular redox balance (Fig. 6b). These findings suggest that the AtGLR3.4.1 receptor may play a critical role in preventing excessive ROS accumulation and maintaining intracellular redox homeostasis under rotenone-induced oxidative stress.

Nicotinamide adenine dinucleotide (NAD) is a crucial metabolite involved in numerous cellular processes, including redox regulation, ATP production, and adaptation to environmental stress. NAD's role in maintaining cellular homeostasis under abiotic stress conditions, particularly mitochondrial stress, has been well-documented (Schwarzländer et al. 2009). NAD plays a central role in controlling the redox status of cells, and its homeostasis is essential for proper stress responses. Disruption of NAD metabolism can have significant consequences on plant stress tolerance, as it directly affects key pathways like the electron transport chain (ETC) and the production of reactive oxygen species (ROS).

In our study, we focused on the role of NAD in the context of rotenone-induced mitochondrial stress. Rotenone, an inhibitor of Complex I in the electron transport system (ETS), causes the accumulation of NADH by inhibiting electron flow, which in turn disrupts the normal ATP production pathway. Under mitochondrial stress conditions, the Col. genotype exhibited an increase in NADH levels, which is consistent with previous studies reporting NADH accumulation under mitochondrial stress (Schwarzländer et al. 2009). However, no such increase in NADH levels was observed in the GLR mutant genotypes. This suggests that alternative NADH-oxidizing enzymes, capable of compensating for the inhibition of Complex I, might have been activated in the mutants, as suggested by Douce and Neuburger (1989). These enzymes may include NADH dehydrogenases or other alternative oxidoreductases that function independently of Complex I, ensuring NADH oxidation and maintaining redox balance under stress conditions. Furthermore, the GLR mutant genotypes displayed a significant decrease in the NAD/NADH ratio compared to the Col. Genotype (Fig. 7c). This indicates increased NAD consumption in these mutants, possibly due to the activation of alternative pathways for NADH oxidation, which leads to a reduction in NAD availability. The significant consumption of NAD may result in a further decrease in the NAD/NADH ratio, which could influence various metabolic pathways and stress response mechanisms. These observations suggest that in GLR mutants, proper NAD utilization and its channeling into the appropriate metabolic pathways are critical for stress tolerance. Disruptions in this process may impair the plants' ability to respond effectively to environmental stress. GLR channels may also play a role in regulating NAD and NADH homeostasis during mitochondrial stress. Mitochondrial stress has a significant impact on NAD/NADH levels, suggesting that this stressor may involve common molecular mechanisms related to NAD metabolism. This highlights the critical role of NAD homeostasis in plant stress responses and indicates that AtGLR3.4 receptors may play an essential role in maintaining NAD balance under mitochondrial stress conditions (Fig. 7c).

Lipid peroxidation (TBARS) and protein oxidation are important indicators of intracellular redox imbalance and oxidative stress, and it has been shown that lipid peroxidation products can increase oxidative modifications in proteins as well as oxidative damage caused by free radicals (Mittler 2002; Levine et al. 1994).

In our study, it was observed that rotenone-induced mitochondrial stress increased TBARS levels. This indicates that cells are exposed to oxidative stress due to mitochondrial dysfunction and increased ROS production. Specifically, the AtGLR3.4.1 gene was found to play a critical role in ROS removal and maintaining cellular redox balance under mitochondrial stress conditions.

The absence of AtGLR3.4.1 led to a significant increase in TBARS levels, while the absence of AtGLR3.4.2 kept this increase at lower levels (Fig. 8a). This suggests that AtGLR3.4.1 plays an important role in controlling oxidative damage within the cell. In *atglr3.4.1* mutants, ROS accumulation increased due to the inhibition of H₂O₂-scavenging enzymes such as catalase (CAT) and ascorbate peroxidase (APX), and the reduction in GSH/GSSG and NAD/NADH ratios led to a disruption in cellular redox balance. These factors explain the higher lipid peroxidation levels observed in *atglr3.4.1* mutants. On the other hand, despite unchanged or decreased antioxidant enzyme activities (SOD, CAT, and POX), the lower TBARS levels in *atglr3.4.2* mutants suggest that these mutants counteract ROS-related oxidative damage through an increase in the activity of ascorbate–glutathione cycle enzymes, and ROS levels may have been regulated through these pathways.

The study of genes associated with mitochondrial stress has an important place in cellular energy production and oxidative phosphorylation processes. In our study, AOX1, UPOX, UCP, SDH2E, COX5b and CI76 (AOX, alternative oxidase; UPOX, upregulated by oxidative stress; UCP, uncoupling protein; SHD2-1, succinate dehydrogenase 2–1 in complex II; COX5b, 5b subunit of cytochrome c oxidase (complex IV); CI76, 76 kDa subunit of complex I) gene expressions that affect these processes and are related to mitochondrial stress were examined. One of the most investigated genes associated with mitochondrial stress is AOX. In plants, in addition to the mitochondrial respiration and cytochrome pathway, there is also an alternative pathway known as AOX (Juszczuk and Rychter 2003). In our study, it was observed that mitochondrial stress increased the transcription of stress-related genes such as AOX1, UPOX, SDH2E, and CI76. The increase in transcription of genes like AOX1 and UPOX suggests the activation of mechanisms that play a crucial role in regulating cellular energy production and oxidative phosphorylation processes. The reduction of these increases in the absence of AtGLR.3.4.1 and AtGLR.3.4.2 indicates that these genes play a role in activating specific defense mechanisms under mitochondrial stress (Figs. 9a, 10a–c).

The absence of AtGLR.3.4.1, which further enhanced the transcriptional increase of AOX1 and UPOX genes, suggests that this receptor plays a critical role in modulating the oxidative stress response. On the other hand, the decrease in these transcriptional increases in the atglr.3.4.2 knockout might indicate that AtGLR.3.4.2 functions through a different mechanism in the regulation of mitochondrial stress. Both AtGLR.3.4.1 and AtGLR.3.4.2 suppressing the transcriptional increase of SDH2E and CI76 suggests that these genes are essential components in mitochondrial energy production and oxidative phosphorylation processes, highlighting the regulatory role of GLR receptors in these processes. CI76 transcription was also increased in all stress conditions and this rise decreased in both atglr.3.4.1 and atglr.3.4.2 knockouts. However, it was at control level in atglr.3.4.2 knockout (Fig. 10a).

The increase in COX5b gene transcription under mitochondrial stress highlights a shift in cellular energy production and oxidative phosphorylation. COX5b, a subunit of the cytochrome c oxidase complex, plays a key role in ATP production (Keunen et. al. 2013). In our study, mitochondrial stress led to increased COX5 activity in Col. plants, indicating a strengthened stress response. Notably, *atglr3.4.1* knockout further enhanced this increase, suggesting that AtGLR3.4.1 regulates COX5b expression during mitochondrial stress. However, in *atglr3.4.2* knockout, COX5b expression remained at control levels, implying that AtGLR3.4.2 modulates stress response through a different mechanism, not directly affecting COX5b transcription (Fig. 9c).

Apart from these genes, uncoupling proteins (UCP) catalyze proton leakage, which disperses the proton electrochemical gradient in the mitochondrial inner membrane. In this way, they shorten the ATP synthase complex and thus inhibit oxidative phosphorylation (Vercesi et al. 2006). In a study, both AOX (Maxwell et al. 1999) and UCP (Pastore et al. 2007) genes were found to reduce mitochondrial ROS formation. Additionally, it was determined that UCP transcription increased under Cd stress (Keunen et. al. 2013) and that the transcription of this gene was increased under mitochondrial stress in mammalian cell lines (Hwang et al. 2014; Hirschenson et al. 2022). In this study, rotenone (R)-induced mitochondrial stress was found to increase UCP transcription, and this increase was further amplified by the knockout of AtGLR.3.4.1. These findings demonstrate the important role of AtGLR3.4 receptors in mitochondrial stress and oxidative stress responses. The increased proton leakage caused by UCP, which impedes ATP production, plays a significant regulatory role under stress conditions that affect mitochondrial energy balance. The knockout of AtGLR3.4.1 further enhanced the increase in UCP transcription, while the knockout of AtGLR3.4.2 did not affect this increase. This suggests that AtGLR3.4.1 plays a regulatory role in strengthening the mitochondrial stress response, while AtGLR3.4.2 may influence this process through a different mechanism (Fig. 9b).

In our study, it was observed that mitochondrial stress increased the transcription of stress-related genes such as AOX1, UPOX, SDH2E, and CI76. The increase in the transcription of genes such as AOX1 and UPOX suggests the activation of mechanisms that play a significant role in regulating cellular energy production and oxidative phosphorylation processes. The reduction in these increases in the absence of AtGLR.3.4.1 and AtGLR.3.4.2 indicates that these genes are involved in activating specific defense mechanisms under mitochondrial stress. The increased transcription of AOX1 and UPOX genes in GLR.3.4.1 knockout suggests that AtGLR.3.4.1 plays a critical role in modulating the oxidative stress response (Figs. 9a, 10b). On the other hand, in atglr.3.4.2 knockout, the reduction of this increase to control levels may suggest that AtGLR.3.4.2 operates through a different mechanism in regulating mitochondrial stress.

Additionally, *atglr3.4* knockout caused a decrease in the expression of AOX, CI7B, and SDH2E genes, while an increase was observed in the expression of UPOX, UCP, and COX5B genes, particularly in the absence of AtGLR3.4.1. Compared to Col., the decrease in AOX1 expression, which encodes proteins and enzymes involved in non-phosphorylated alternative mitochondrial respiratory pathways, and the decrease in Complex I and Complex II gene expressions suggest the importance of AtGLR3.4 receptors in the activation of this pathway. Conversely, the higher expression of Complex IV, UPOX, and UCP genes in mutants under stress indicates the sensitivity of these genes to *atglr3.4* mutants and suggests that these receptors play a decisive role in regulating the mitochondrial stress response.

## Conclusion

This study examined the roles of AtGLR3.4.1 and AtGLR3.4.2 genes in mitochondrial stress, particularly in ROS regulation and stress-related gene expression in plants. The knockout of AtGLR3.4.1 increased ROS accumulation, likely due to the inhibition of H₂O₂-scavenging enzymes such as CAT and APX, as well as a decrease in GSH/GSSG and NAD/NADH ratios, disrupting cellular redox balance. Additionally, *atglr3.4.1* knockout supported the transcription of stress-related genes like COX5B, UPOX, and UCP1, suggesting its role in enhancing mitochondrial stress responses and triggering antioxidant defense mechanisms. In contrast, *atglr3.4.2* mutants appeared to counteract ROS-related oxidative damage by increasing the activity of ascorbate–glutathione cycle enzymes, potentially regulating ROS levels through this pathway. These findings highlight the critical role of AtGLR3.4 receptors in ROS regulation and mitochondrial stress responses, suggesting their potential for biotechnological applications to enhance plant stress tolerance. Our findings highlight the important role of AtGLR3.4 in NO-related redox signaling and mitochondrial stress responses in Arabidopsis, without aiming to draw direct parallels to mammalian systems.

## Data Availability

All data generated or analysed during this study are included in this published article.
